# Associations of social interactions during the COVID-19 pandemic with cognitive function among the South Korean older adults

**DOI:** 10.1186/s12877-023-04112-9

**Published:** 2023-06-28

**Authors:** Il Yun, Yu Shin Park, Eun-Cheol Park, Hee-Won Jung, Jaeyong Shin

**Affiliations:** 1 Department of Public Health, Graduate School, Yonsei University, Seoul, Republic of Korea; 2 Institute of Health Services Research, Yonsei University, Seoul, Republic of Korea; 3 Department of Preventive Medicine, Yonsei University College of Medicine, 50-1 Yonsei-to, Seodaemun-Gu, Seoul, 03722 Republic of Korea; 4 Division of Geriatrics, Asan Medical Center, Seoul, Republic of Korea

**Keywords:** Cognitive function, South Korea, Social interaction, Social distancing, COVID-19 pandemic

## Abstract

**Background:**

We aimed to demonstrate the associations between social interactions within social distancing norms during the coronavirus disease 2019 (COVID-19) pandemic and cognitive function among South Korean older adults.

**Methods:**

Data from the 2017 and 2020 Survey of Living Conditions and Welfare Needs of Korean Older Persons were used. There were 18,813 participants (7,539 males; 11,274 females). T-test and multiple logistic regression analyses verified whether the mean difference in older adults’ cognitive function before and during the COVID-19 pandemic was statistically significant. We also examined the associations between social interactions and cognitive function. The key results were presented as odds ratios (ORs) and 95% confidence intervals (CI).

**Results:**

All participants were more likely to experience cognitive impairment during the COVID-19 pandemic than before (males: OR 1.56, 95% CI 1.3–1.78; females: OR 1.26, 95% CI: 1.14–1.40). Cognitive impairment increased linearly with the decreased frequency of face-to-face contact with non-cohabiting children. Possible cognitive impairment was greater for females who had not visited senior welfare centers for the past year (OR 1.43, 95% CI 1.21–1.69).

**Conclusion:**

Korean older adults’ cognitive function declined during the COVID-19 pandemic and was associated with reduced social interactions because of social distancing measures. Alternative interventions should be promoted for safely restoring social networks, considering the adverse effects of long-term social distancing on older adults’ mental health and cognitive function.

## Introduction

The worldwide spread of coronavirus disease (COVID-19) and the resulting deaths caused the World Health Organization to declare a global pandemic on March 12, 2020 [1]. Since the first COVID-19 case was reported in South Korea on January 20, 2020, the number of confirmed cases have increased continuously in the past three years [2]. As of February 2023, the cumulative number of confirmed cases in Korea had reached 30 million, and 3 out of 5 people in the entire population were infected.

The Korean government has ardently promoted various public health interventions to prevent transmission, including public campaigns for social distancing, early detection testing, isolation and quarantine measures, and contact tracing. In addition, Korean citizens were instructed to practice personal hygiene, such as wearing masks indoors and outdoors and avoiding private gatherings [2, 3]. These non-pharmaceutical interventions could help control the spread of the virus; however, implementing a long-term social distancing policy faced many challenges; for instance, it incurred higher socio-economic costs [3, 4]. Therefore, the Korean government has declared a new blueprint, “Living with COVID-19” known as “With Corona” to prepare for a gradual return to normal life, and the first phase began on November 1, 2021.

Prolonged social distancing mitigated disease spread effectively and minimized its critical impact on older adults [5]. However, there was a concern that it would adversely affect their mental health; for instance, the lack of social interactions would cause loneliness and isolation [6]. As is well documented, social isolation harms the older adult’s quality of life as well as their physical and mental health [7], and limited social interactions are associated with psychological distress and cognitive deficits among the older people [8–10].

However, in Korea, few prior studies have examined the association between social interactions and cognitive function of older adults during the COVID-19 pandemic. Therefore, this study aimed to demonstrate the associations between cognitive function and reduced social interactions, in compliance with social distancing measures during the COVID-19 pandemic, among older South Koreans.

## Materials and methods

### Data

This study used data obtained from the 2017 and 2020 Survey of Living Conditions and Welfare Needs of Korean Older Persons, conducted by the Korean Institute for Health and Social Welfare. This nationwide survey was based on the Elderly Welfare Act, and since 2008, five surveys have been conducted every three years [11, 12]. The question used in each survey are the same, and data collection methods have not changed during the pandemic.

The purpose of this data collection was to provide a basis for establishing welfare policies to improve the older adults’ quality of life, examine changes in their characteristics through time-series data accumulation, and respond to the older population. Since all participants provided informed consent in advance, and the data were publicly accessible, no further ethical approval was required.

### Study population

The survey population from the recent two waves (2017 and 2020) included 20,396 individuals. Among the population for this study, we selected the older adults who currently live separately from their children to confirm the impact of decreased face-to-face contact with their non-cohabiting children, in compliance with social distancing measures. After excluding missing data (*N* = 180), responses from 18,813 participants (7,539 males; 11,274 females) comprised the study sample.

### Variables

The dependent variable was cognitive function, assessed using the Korean version of the Mini-Mental State Examination for Dementia Screening (MMSE-DS) [13]. A binary variable was formed based on the criteria that any score over 24 (out of 30) indicates normal cognition, and a score less than 24 is considered cognitive impairment [14, 15]. The main independent variables were the outbreak of the COVID-19 pandemic and the participant’s social interactions. The COVID-19 outbreak variable was classified into two waves: before the COVID-19 pandemic in 2017 and during the COVID-19 pandemic in 2020. The social interactions variable, defined as social behavior patterns that occur when complying with social distancing norms during the pandemic, includes the following three variables: face-to-face contact with their non-cohabiting children (seldom:1 ~ 2 times a year, occasionally: 1 ~ 2 times per quarter, frequently: at least once a week), visiting senior community centers (yes or no), and visiting senior welfare centers (yes or no).

Data regarding socio-demographic factors and health-related variables, potential confounders, included gender, age (65–69, 70–74, 75–79 and 80 or over), region (urban and rural), marital status (unmarried or separated and married), and schooling years (0 ~ 6, 7 ~ 12, and 13 or over). In addition, economic activity was classified based on whether the participants were currently economically active or inactive. Variables regarding health behavior patterns such as drinking, smoking, and physical activity were also considered. Furthermore, experiences of dementia and depression diagnoses from doctors were also corrected.

### Statistical analysis

The chi-squared test was conducted to compare the general characteristics of the study population. Subsequently, t-test was performed to verify whether the mean difference in the older adult’s cognitive function between the two waves (before and during the COVID-19 pandemic) was statistically significant. Multiple logistic regression was conducted to examine the associations between social interactions that comply with social distancing norms during the pandemic and cognitive function among older Korean adults. The key results were presented as odds ratios (ORs) and 95% confidence intervals (CI). For all analyses, we used SAS version 9.4 (SAS Institute Inc; Cary, NC, USA), and *p*-values less than 0.01 were considered statistically significant.

## Results

Table 1 presents the general characteristics of the study population stratified by gender, in which 29.1% of men (*N* = 2,193) and 46.7% of women (*N* = 5,268) were identified as cognitively impaired with an MMSE-DS score of less than 24. After the outbreak of the COVID-19 pandemic, the proportion of the participants with cognitive impairment was higher for both men and women. Furthermore, based on the t-test results, the mean difference in MMSE-DS scores between the two waves (the period before and during the COVID-19 pandemic) was found to be statistically significant (*p* < 0.0001).

**Table 1:** General characteristics of the study population

Variables	Male							Female						
	**Cognitive impairment**							**Cognitive impairment**						
	**Total**		**Yes**		**No**		***P*** **-value**	**Total**		**Yes**		**No**		***P*** **-value**
	**N**	**%**	**N**	**%**	**N**	**%**		**N**	**%**	**N**	**%**	**N**	**%**	
**Total (** ***N*** ** = 18,813)**	**7,539**	**100.0**	**2,193**	**29.1**	**5,346**	**70.9**		**11,274**	**100.0**	**5,268**	**46.7**	**6,006**	**53.3**	
Outbreak of COVID-19							< .0001							< .0001
2017 (Before the COVID-19)	3,815	50.6	968	25.4	2,847	74.6		5,706	50.6	2,610	45.7	3,096	54.3	
2020 (During the COVID-19)	3,624	48.1	1,125	31.0	2,499	69.0		5,568	49.4	2,658	47.7	2,910	52.3	
Face-to-face contact with their noncohabiting children							0.0003							0.0003
Seldom	930	12.3	322	34.6	608	65.4		1,311	11.6	672	51.3	639	48.7	
Occasionally	4,558	60.5	1,308	28.7	3,250	71.3		6,746	59.8	3,158	46.8	3,588	53.2	
Frequently	2,051	27.2	563	27.5	1,488	72.5		3,217	28.5	1,438	44.7	1,779	55.3	
Visiting the senior community center							< .0001							< .0001
Yes	1,726	22.9	695	40.3	1,031	59.7		3,796	33.7	2,356	62.1	1,440	37.9	
No	5,813	77.1	1,498	25.8	4,315	74.2		7,478	66.3	2,912	38.9	4,566	61.1	
Visiting the senior welfare center							0.798							0.0002
Yes	623	8.3	184	29.5	439	70.5		1,056	9.4	435	41.2	621	58.8	
No	6,916	91.7	2,009	29.0	4,907	71.0		10,218	90.6	4,833	47.3	5,385	52.7	
Age							< .0001							< .0001
65 ~ 69	2,281	30.3	404	17.7	1,877	82.3		3,360	29.8	847	25.2	2,513	74.8	
70 ~ 74	2,052	27.2	504	24.6	1,548	75.4		2,775	24.6	1,147	41.3	1,628	58.7	
75 ~ 79	1,765	23.4	602	34.1	1,163	65.9		2,581	22.9	1,461	56.6	1,120	43.4	
80 or over	1,441	19.1	683	47.4	758	52.6		2,558	22.7	1,813	70.9	745	29.1	
Region							< .0001							< .0001
Urban	3,029	40.2	793	26.2	2,236	73.8		4,546	40.3	1,911	42.0	2,635	58.0	
Rural	4,510	59.8	1,400	31.0	3,110	69.0		6,728	59.7	3,357	49.9	3,371	50.1	
Marital status							< .0001							< .0001
Unmarried or Being separately	1,121	14.9	396	35.3	725	64.7		6,003	53.2	3,334	55.5	2,669	44.5	
Married	6,418	85.1	1,797	28.0	4,621	72.0		5,271	46.8	1,934	36.7	3,337	63.3	
Schooling years							< .0001							< .0001
0 ~ 6	2,564	34.0	1,123	43.8	1,441	56.2		7,285	64.6	4,293	58.9	2,992	41.1	
7 ~ 12	4,105	54.5	959	23.4	3,146	76.6		3,677	32.6	928	25.2	2,749	74.8	
13 or over	870	11.5	111	12.8	759	87.2		312	2.8	47	15.1	265	84.9	
Economic activity							< .0001							< .0001
Yes	3,314	44.0	807	24.4	2,507	75.6		3,321	29.5	1,324	39.9	1,997	60.1	
No	4,225	56.0	1,386	32.8	2,839	67.2		7,953	70.5	3,944	49.6	4,009	50.4	
Smoking							< .0001							0.420
Yes	1,581	21.0	393	24.9	1,188	75.1		273	2.4	121	44.3	152	55.7	
No	5,958	79.0	1,800	30.2	4,158	69.8		11,001	97.6	5,147	46.8	5,854	53.2	
Drinking							< .0001							< .0001
Seldom	3,888	51.6	1,294	33.3	2,594	66.7		9,904	87.8	4,727	47.7	5,177	52.3	
Occasionally	2,185	29.0	542	24.8	1,643	75.2		1,156	10.3	448	38.8	708	61.2	
Frequently	1,466	19.4	357	24.4	1,109	75.6		214	1.9	93	43.5	121	56.5	
Physical exercise							< .0001							< .0001
Yes	4,793	63.6	1,174	24.5	3,619	75.5		6,506	57.7	2,696	41.4	3,810	58.6	
No	2,746	36.4	1,019	37.1	1,727	62.9		4,768	42.3	2,572	53.9	2,196	46.1	
Dementia checkup							0.740							0.512
Yes	2,883	38.2	845	29.3	2,038	70.7		5,050	44.8	2,377	47.1	2,673	52.9	
No	4,656	61.8	1,348	29.0	3,308	71.0		6,224	55.2	2,891	46.4	3,333	53.6	
Diagnosed with Depression							0.719							0.304
Yes	70	0.9	19	27.1	51	72.9		374	3.3	165	44.1	209	55.9	
No	7,469	99.1	2,174	29.1	5,295	70.9		10,900	96.7	5,103	46.8	5,797	53.2	

**Fig. 1:**
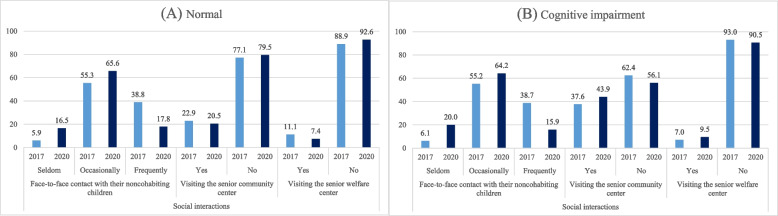
Changes in social interactions of the Korean older adults before and during the COVID-19 pandemic

Figure 1 illustrates the frequency of change in the participants’ social interactions and the difference between 2017 and 2020. The biggest change was in the frequency of face-to-face contact with their non-cohabiting children. Compared to the period before the COVID-19 outbreak in 2017, the percentage of frequent contact decreased significantly during the pandemic. The percentage of individuals who have frequent contact with their children, at least once a week, has decreased significantly since the pandemic among both those with normal cognitive function (-21.0%p) and those with cognitive impairment(-22.8%p).

As noted in Table 2, the associations between social interactions and cognitive function among older Korean adults were identified. Both men and women were more likely to be cognitively impaired during the COVID-19 pandemic, compared to before the pandemic (Men: OR 1.56, 95% CI 1.36–1.78; Women: OR 1.26, 95% CI: 1.14–1.40). The ORs increased linearly as the frequency of face-to-face contact with the participants’ non-cohabiting children decreased. When a "frequent contact” was set as the reference category, statistical significance was found for “seldom contact” for men (OR 1.34, 95% CI 1.10–1.64), and “occasional” and “seldom contact” for women (Occasional contact: OR 1.19, 95% CI 1.07–1.33; Seldom contact: OR 1.43, 95% CI 1.12–1.68). The association between visits to senior community centers and cognitive function was not identified; however, the possibility of cognitive impairment was higher in older women who had not visited senior welfare centers in the past year than it was for those older women who had (OR 1.43, 95% CI 1.21–1.69).

**Table 2:** Results of factors associated with cognitive function

Variables	Male		Female	
	**Cognitive impairment**		**Cognitive impairment**	
	**OR**	**95% CI**	**OR**	**95% CI**
Outbreak of COVID-19				
2017 (Before the COVID-19)	1.00		1.00	
2020 (During the COVID-19)	1.56 ^*^	(1.36-1.78)	1.26 ^*^	(1.14-1.40)
Face-to-face contact with their noncohabiting children				
Seldom	1.34 ^*^	(1.10-1.64)	1.43 ^*^	(1.21-1.68)
Occasionally	1.04	(0.90-1.19)	1.19 ^*^	(1.07-1.33)
Frequently	1.00		1.00	
Visiting the senior community center				
Yes	1.00		1.00	
No	0.71 ^*^	(0.61-0.83)	0.57 ^*^	(0.51-0.64)
Visiting the senior welfare center				
Yes	1.00		1.00	
No	1.13	(0.90-1.42)	1.43 ^*^	(1.21-1.69)
Age				
65 ~ 69	1.00		1.00	
70 ~ 74	1.25 ^*^	(1.06-1.49)	1.57 ^*^	(1.38-1.79)
75 ~ 79	1.82 ^*^	(1.53-2.18)	2.27 ^*^	(1.96-2.63)
80 or over	2.78 ^*^	(2.28-3.38)	3.44 ^*^	(2.94-4.02)
Region				
Urban	1.00		1.00	
Rural	1.09	(0.96-1.25)	1.07	(0.97-1.19)
Marital status				
Unmarried or Being separately	1.00	(0.85-1.18)	1.23 ^*^	(1.11-1.37)
Married	1.00		1.00	
Schooling years				
0 ~ 6	3.86 ^*^	(3.01-4.94)	4.49 ^*^	(3.07-6.56)
7 ~ 12	2.02	(1.59-2.58)	1.93	(1.32-2.84)
13 or over	1.00		1.00	
Economic activity				
Yes	1.00		1.00	
No	1.39 ^*^	(1.22-1.59)	1.31 ^*^	(1.18-1.47)
Smoking				
Yes	0.92	(0.78-1.07)	0.91	(0.67-1.24)
No	1.00		1.00	
Drinking				
Seldom	1.00		1.00	
Occasionally	0.82	(0.71-0.94)	0.86	(0.73-1.01)
Frequently	0.75 ^*^	(0.63-0.88)	0.90	(0.65-1.26)
Physical exercise				
Yes	1.00		1.00	
No	1.58 ^*^	(1.39-1.79)	1.32 ^*^	(1.20-1.46)
Dementia checkup				
Yes	1.00		1.00	
No	1.17	(1.03-1.33)	1.22 ^*^	(1.11-1.35)
Diagnosed with Depression				
Yes	0.71	(0.37-1.33)	1.08	(0.82-1.42)
No	1.00		1.00	

ORs were adjusted for all covariates

^*^ *P*-value < 0.01

**Fig. 2:**
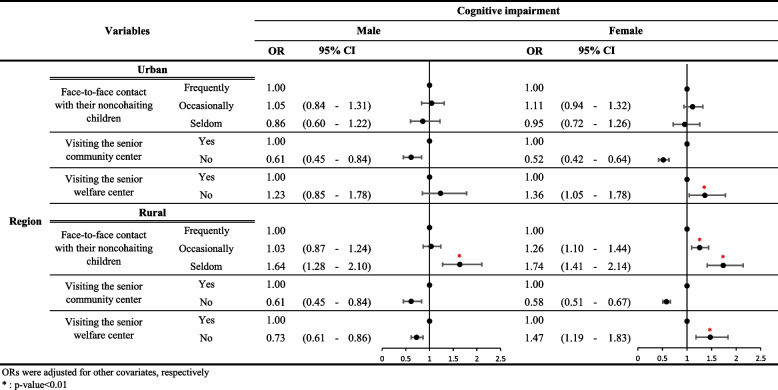
Results of subgroup analysis stratified by region

We also conducted subgroup analysis stratified by region; Fig. 2 illustrates the results. For example, visits to senior welfare centers in the past year were associated with urban residents’ cognitive impairment. Conversely, a significant change in ORs of cognitive impairment was confirmed for rural residents according to face-to-face contact frequency with their non-cohabiting children.

## Discussion

This study presents the following three key findings. First, the mean of the MMSE-DS score, a measure of the prevalence of cognitive impairment, was lower during, compared to before, the COVID-19 pandemic. The MMSE-DS score of the study population before the pandemic was 25.2 ± 5.2 (mean ± SD; median: 26.0) out of 30, and the MMSE-DS score tested during the pandemic was 24.6 ± 6.9 (mean ± SD; median: 25.0) out of 30. Second, changes in social interactions among the older adults before and during the pandemic were noted and are attributed to the impact of strong social distancing measures. The decline in social interactions during the pandemic were relatively greater, especially in the cognitively impaired group. Third, the participants’ social interactions were associated with their cognitive function. This association was investigated particularly among residents in rural areas.

The core results of our study were similar to those in previous studies examining the impacts of social networks, social interactions, social ties, and social isolation on the quality of life of older adults and their mental health, including depression [16–19], loneliness [20, 21], suicidal ideation [22–24], and cognitive function [25]. Many of studies on the health and well-being of older adults have also demonstrated this association [26, 27]; however, few studies have considered the impact of COVID-19 using recent data. Therefore, this study is meaningful in that we used the most recent time-series survey data performed both before and after the COVID-19 outbreak to investigate and compare the impact of social distancing norms during the pandemic on the social interactions among the older Korean adults, and the possibility of cognitive impairment due to reduced social interactions.

Korea has the highest suicide rate of older adults among OECD countries. In addition, Alzheimer’s disease is the seventh leading cause of death, and mental health problems were recognized as a critical phenomenon affecting the mortality of the older population [28]. With this background, we could provide some public health implications by reflecting on current social issues related to the mental health of older Koreans. Amid the crisis caused by the pandemic, the effect of social distancing on COVID-19 incidence and mortality has been proven [29]. However, such interventions can also have adverse effects; for instance, they cause changes in social networks and daily lifestyles. Therefore, social distancing and social support interventions should be implemented simultaneously for socially vulnerable groups such as older individuals. For instance, it would be helpful to provide telehealth services to vulnerable groups suffering from deterioration of physical and mental health [30].

Furthermore, due to the prolonged COVID-19 pandemic, most of our social networks are expected to include non-face-to-face communication. Thus, older adults must become accustomed to non-face-to-face interactions, such as video meetings and online classes. In many countries including Korea, job training, leisure activities, and e-learning programs for persons 65 years and older previously held at senior welfare centers, are being implemented in a non-face-to-face system.

This study had certain limitations. First, the data were based on self-reports; hence, the interactions may not have been accurately measured and may be less reliable. It may not be easy for older adults to fully recall their social interactions over the past year, especially those with cognitive impairment. Second, we included only three types of social behavior patterns as the social interactions: face-to-face contact with their non-cohabiting children and visits to senior community centers and welfare center; however, more types of social interactions may be included in future studies. Furthermore, the data excluded the frequency of contact with relatives and friends, which may have decreased during the pandemic. Third, although we attempted to control for numerous covariates that may affect the dependent variable, residual confounding effects from unmeasured variables could not be ruled out.

## Conclusion

This study demonstrated that cognitive decline in older Korean adults during the COVID-19 pandemic was associated with reduced social interactions in compliance with social distancing norms. While social distancing effectively reduced the incidence of and mortality associated with COVID-19, it may have adversely affected older adults’ mental health and cognitive function. Thus, alternative interventions should be promoted to safely restore the social networks during the “With Corona”.

## Data Availability

The data is publicly accessible and can be shared through application on the website of Korea Institute for Health and Social Affairs (https://data.kihasa.re.kr/kihasa/kor/contents/ContentsList.html).

## References

[1] Ciotti M, Ciccozzi M, Terrinoni A, Jiang W-C, Wang C-B, Bernardini S (2020). The COVID-19 pandemic. Crit Rev Clin Lab Sci.

[2] Choe YJ, Lee J-K (2020). The impact of social distancing on the transmission of influenza virus, South Korea, 2020. Osong Pub Health Res Perspect.

[3] Kim S, Ko Y, Kim Y-J, Jung E (2020). The impact of social distancing and public behavior changes on COVID-19 transmission dynamics in the Republic of Korea. PLoS ONE.

[4] Kim E-A (2020). Social distancing and public health guidelines at workplaces in Korea: responses to coronavirus disease-19. Saf Health Work.

[5] Van Orden KA, Bower E, Lutz J, Silva C, Gallegos AM, Podgorski CA, Santos EJ, Conwell Y (2021). Strategies to promote social connections among older adults during “social distancing” restrictions. Am J Geriatr Psychiatry.

[6] Aleman A, Sommer I (2020). The silent danger of social distancing. Psychol Med.

[7] Leigh-Hunt N, Bagguley D, Bash K, Turner V, Turnbull S, Valtorta N, Caan W (2017). An overview of systematic reviews on the public health consequences of social isolation and loneliness. Public Health.

[8] Evans IE, Martyr A, Collins R, Brayne C, Clare L (2019). Social isolation and cognitive function in later life: a systematic review and meta-analysis. J Alzheimers Dis.

[9] Fu C, Li Z, Mao Z (2018). Association between social activities and cognitive function among the elderly in China: a cross-sectional study. Int J Environ Res Public Health.

[10] Li HO-Y, Huynh D (2020). Long-term social distancing during COVID-19: A social isolation crisis among seniors?. CMAJ.

[11] Lee S-Y, Atteraya MS (2019). Depression, poverty, and abuse experience in suicide ideation among older Koreans. Int J Aging Human Dev.

[12] Baek JY, Lee E, Jung H-W, Jang I-Y (2021). Geriatrics fact sheet in Korea 2021. Ann Geriatric Med Res.

[13] Oh E, Kang Y, Shin J, Yeon B (2010). A validity study of K-MMSE as a screening test for dementia: comparison against a comprehensive neuropsychological evaluation. Dement Neurocog Disord.

[14] Crum RM, Anthony JC, Bassett SS, Folstein MF (1993). Population-based norms for the mini-mental state examination by age and educational level. JAMA.

[15] Nari F, Jeong W, Jang BN, Lee HJ, Park E-C (2021). Association between healthy lifestyle score changes and quality of life and health-related quality of life: a longitudinal analysis of South Korean panel data. BMJ Open.

[16] Mair CA (2010). Social ties and depression: an intersectional examination of Black and White community-dwelling older adults. J Appl Gerontol.

[17] Blazer DG (1983). Impact of late-life depression on the social network. Am J Psychiatry.

[18] Domènech-Abella J, Lara E, Rubio-Valera M, Olaya B, Moneta MV, Rico-Uribe LA, Ayuso-Mateos JL, Mundó J, Haro JM (2017). Loneliness and depression in the elderly: the role of social network. Soc Psychiatry Psychiatr Epidemiol.

[19] Rosenquist JN, Fowler JH, Christakis NA (2011). Social network determinants of depression. Mol Psychiatry.

[20] Coyle CE, Dugan E (2012). Social isolation, loneliness and health among older adults. J Aging Health.

[21] Kemperman A, van den Berg P, Weijs-Perrée M, Uijtdewillegen K (2019). Loneliness of older adults: social network and the living environment. Int J Environ Res Public Health.

[22] Heuser C, Howe J. The relation between social isolation and increasing suicide rates in the elderly. Qual Ageing Older Adults. 2019;20(1):2-9.

[23] Dsir TO (2017). Suicide among the elderly in Korea: a meta-analysis. Innov Aging.

[24] Mireault M, De Man AF (1996). Suicidal ideation among the elderly: Personal variables, stress and social support. Soc Behav Personal Int J.

[25] Siette J, Georgiou A, Brayne C, Westbrook JI (2020). Social networks and cognitive function in older adults receiving home-and community-based aged care. Arch Gerontol Geriatr.

[26] Park S, Kim TH, Eom TR (2021). Impact of social network size and contact frequency on resilience in community-dwelling healthy older adults living alone in the Republic of Korea. Int J Environ Res Public Health.

[27] Han S, Kim H, Lee H-S (2020). Social Capital and Its Association with Health and Well-Being.

[28] Korea S (2021). Cause of death statistics in the Republic Korea, 2019.

[29] Thu TPB, Ngoc PNH, Hai NM (2020). Effect of the social distancing measures on the spread of COVID-19 in 10 highly infected countries. Sci Total Environ.

[30] Fisk M, Livingstone A, Pit SW (2020). Telehealth in the context of COVID-19: changing perspectives in Australia, the United Kingdom, and the United States. J Med Internet Res.

